# Patient and Provider Experiences With a Digital App to Improve Compliance With Enhanced Recovery After Surgery (ERAS) Protocols: Mixed Methods Evaluation of a Canadian Experience

**DOI:** 10.2196/49277

**Published:** 2023-12-15

**Authors:** Sanjay Beesoon, Ashley Drobot, Melissa Smokeyday, Al-Bakir Ali, Zoe Collins, Colin Reynolds, Sandra Berzins, Alison Gibson, Gregg Nelson

**Affiliations:** 1 Surgery Strategic Clinical Network Alberta Health Services Edmonton, AB Canada; 2 Faculty of Medicine and Dentistry University of Alberta Edmonton, AB Canada; 3 Health Systems Knowledge and Evaluation Alberta Health Services Edmonton, AB Canada; 4 Department of Community Health Sciences Cumming School of Medicine University of Calgary Calgary, AB Canada; 5 Okanagan College Community Engagement and Careers Okanagan, BC Canada; 6 Department of Obstetrics & Gynecology Cumming School of Medicine University of Calgary Calgary, AB Canada

**Keywords:** app, digital health, patient experience, provider satisfaction, application, recovery, cost-effective, evaluation, implementation, gynecologic oncology, colorectal surgery, surgery, care

## Abstract

**Background:**

Of all the care provided in health care systems, major surgical interventions are the costliest and can carry significant risks. Enhanced Recovery After Surgery (ERAS) is a bundle of interventions that help improve patient outcomes and experience along their surgical journey. However, given that patients can be overwhelmed by the multiple tasks that they are expected to follow, a digital application, the ERAS app, was developed to help improve the implementation of ERAS.

**Objective:**

The objective of this work was to conduct a thorough assessment of patient and provider experiences using the ERAS app.

**Methods:**

Patients undergoing colorectal or gynecological oncology surgery at 2 different hospitals in the province of Alberta, Canada, were invited to use the ERAS app and report on their experiences using it. Likewise, care providers were recruited to participate in this study to provide feedback on the performance of this app. Data were collected by an online survey and using qualitative interviews with participants. NVivo was used to analyze qualitative interview data, while quantitative data were analyzed using Excel and SPSS.

**Results:**

Overall, patients found the app to be helpful in preparation for and recovery after surgery. Patients reported having access to reliable unbiased information regarding their surgery, and the app provided them with clarity of actions needed along their surgical journey and enhanced the self-management of their care. Clinicians found that the ERAS app was easy to navigate, was simple for older adults, and has the potential to decrease unnecessary visits and phone calls to care providers. Overall, this proof-of-concept study on the use of a digital health app to accompany patients during their health care journey has shown positive results.

**Conclusions:**

This is an important finding considering the massive investment and interest in promoting digital health in health care systems around the world.

## Introduction

By the very nature of its invasiveness and the risks associated with it, surgery weighs heavily on the overall health care expenditure. Thus, significant investments are being made to improve the quality and safety of surgical care with the downstream effect of decreasing complications, reducing length of stay, and increasing patient satisfaction [1,2]. Enhanced Recovery After Surgery (ERAS) is an innovative program that incorporates multimodal data-driven interventions targeting patients before, during, and after surgery [3]. These interventions are delivered by a multidisciplinary team of health care professionals (eg, surgeons, anesthesiologists, nurses) and include carbohydrate fluid loading prior to surgery, intraoperative warming, early mobilization, and avoidance of opioids, among several other interventions [4].

There is consistent reliable evidence that implementation of ERAS programs in multiple surgical specialties have led to lower incidence of postoperative complications [5-7], thus leading to lower lengths of stay [6,8,9] and readmission rates [10], lower surgical infection rates [11], improved overall patient satisfaction [12], reduced health care utilization, and decreased hospital costs [13]. All these benefits are contingent upon steady, strict, and sustained adherence with the ERAS protocols by both health care providers and patients. Health care professionals, given clear clinical guidelines and with accountability for their professional conduct, generally adhere to and document standard protocols. However, this is not always the case for patients, who, for a variety of reasons, do not follow established patient guidelines in a rigorous and consistent manner. The ERAS program is a partnership between health care professionals and patients. Its success is heavily dependent on patients’ knowledge of and attitudes toward the program and their ability to follow the protocols before, during, and after surgery [14]. Understanding and addressing these patient-related factors are crucial for the ongoing development and optimization of the ERAS program.

There is no doubt that, over the past decade, there has been an explosion in digital health technologies, including mobile health apps. A recently published study revealed that more than 90,000 digital health apps were added to app stores in 2020, which is about 250 apps added every day [15]. Many of these apps are co-designed and developed by software engineers and health care professionals in direct response to market demand, with the primary objectives being to improve patient outcomes, patient experience, and overall health system performance. However, some digital health apps are simply driven by industry, with profit as the primary objective. In this complex and rapidly evolving digital health landscape, health system research is needed to assess the effectiveness of these digital tools to make a meaningful and measurable difference in clinical outcomes and patient and provider satisfaction in specific populations prior to promoting their use [16].

A recent study showed that patients who received ERAS care within Alberta Health Services (AHS), expressed a need for more information and education about pre and postoperative care [14,17]. In response to the patient recommendations identified, a digital health patient app (ERAS app) was developed and customized for AHS, the single health authority in the province of Alberta, Canada.

**Figure 1:**
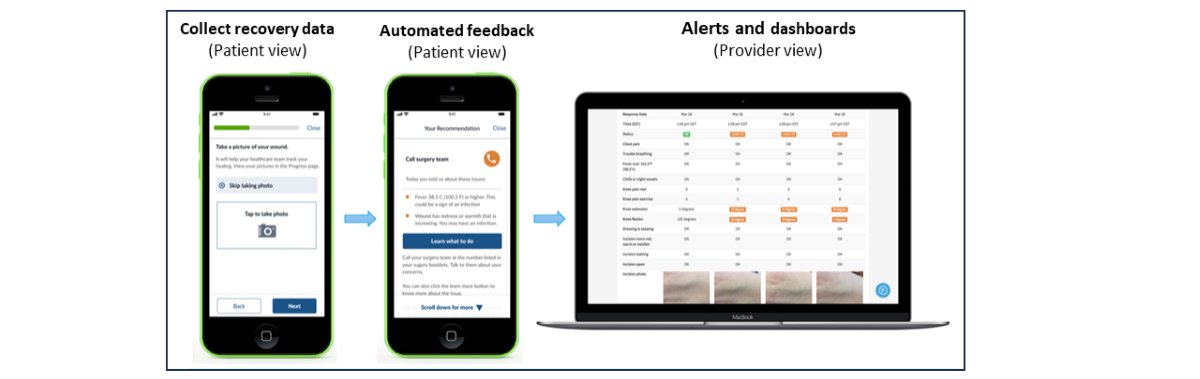
Patient and provider views of the Enhanced Recovery After Surgery (ERAS) app.

The ERAS app used in this study supports patients with tailored education and activities through their preoperative preparation, in-hospital recovery, and postdischarge care. The ERAS app provides patients with information on “What to Expect” during each surgical phase, an extensive “Education Library,” and a postoperative “Daily Health Check Questionnaire,” which provides patients with feedback and allows them to track their progress. The ERAS app also has “To-Do Lists” and “What is Next” in their surgical journey and daily reminder features to help with patient scheduling. The patient and provider views of the app (used with permission from Seamless MD) are provided in Figure 1.

The main objective of this study was to evaluate the implementation of the ERAS app, with an emphasis on patient and provider experiences in gynecologic oncology (GO) and colorectal (CR) surgery.

## Methods

### Setting

After feasibility pilot testing and app deployment for 24 months within 2 surgical centers, the phased evaluation study began. The first phase of the study involved customizing an existing ERAS web and mobile app (SeamlessMD) to reflect Alberta content. This phase provided limited feasibility testing at 2 ERAS sites and patients undergoing 1 of 2 ERAS surgeries (ERAS GO patients at Foothills Medical Centre [FMC] in Calgary and ERAS CR patients at Grey Nuns Community Hospital [GNCH], Covenant Health in Edmonton). The second phase involved expanded recruitment over 7 months to all FMC GO and GNCH CR ERAS patients with evaluation of impacts for patients and clinicians.

### Study Design

The evaluation used a mixed methods approach, collecting both quantitative survey and qualitative interview data, to understand the experiences and satisfaction of patients and clinicians who used the app. Qualitative data were organized using NVivo 11 software, and both Microsoft Excel and SPSS (Version 23.0; IBM Corp) were used to analyze the quantitative data. The development of the survey tools was a collaborative multidisciplinary team effort by surgeons, epidemiologists, statisticians, evaluation consultants, qualitative researchers, and a software engineer. The consent form (Multimedia Appendix 1), interview guides (Multimedia Appendix 2), and surveys (Multimedia Appendix 3) are available in the multimedia appendices.

### Recruitment

Patient recruitment for the ERAS app was promoted through various posters and handouts at presurgery clinic visits to patient candidates for GO surgery or CR surgery using ERAS protocols at the FMC and GNCH, respectively. As part of the intervention arm (ERAS APP) of the study, patients self-enrolled in the study between December 2018 and June 2019 and received access to the ERAS app via a home computer or mobile device. Patients undergoing ERAS surgery at 2 additional hospital sites, Royal Alexandra Hospital Edmonton Zone for GO non-ERAS app patients and Peter Lougheed Centre Calgary Zone for CR non-ERAS app patients, served as nonintervention controls.

### Patient Experience

To understand patient experiences with the ERAS app, 18 patients who used the ERAS app were invited to participate in a 15-minute telephone interview to discuss their experiences using the ERAS app. Patients were contacted up to 6 times (3 daytime attempts and 3 evening attempts) to participate in an interview. If no telephone numbers were available, patients were contacted via email. The 4 major themes of patient interviews were the (1) overall experience using the app, (2) experience with the recruitment and sign-on process, (3) views on strengths or weaknesses of the app, and (4) suggestions to improve the app. In accordance with the research protocol, patients were informed that participation was voluntary, confidentiality of personal information would be maintained, and all interview audio recordings would be destroyed at project end. With participant consent, interviews were audio-recorded, anonymized, and transcribed for analysis. Responses were themed using an open coding technique in NVivo. The ERAS app included a patient satisfaction questionnaire to assess users’ overall satisfaction with accessing the app, using the app, the app functionality, and content within the app. Responses were analyzed and themed with the patient interview data.

### Clinician Experience

We invited 8 clinicians who were involved in ERAS app patient recruitment to participate in a 15-minute to 30-minute telephone interview to discuss their experiences with the ERAS app. Clinician experience with the ERAS app, strengths and weaknesses, comments or recommendations for future implementation, and thoughts about expansion of the ERAS app were gathered via telephone interviews. The focus of the interviews was on (1) overall experience with the ERAS app pilot, (2) views on strengths or weaknesses of the app, (3) comments or recommendations for future implementations, and (4) recommendation for the app to be continued or expanded. The same protocol of audio recording, anonymizing, and transcription used for patient interviews was used for clinician interviews.

### Surveys

The Quality of Recovery-15 (QoR-15) survey was offered 14 days after GO and CR surgeries to assess how the patients felt in the last 24 hours (Part A: 10 questions) and if they felt pain, nausea, anxiety, or depression in the last 24 hours (Part B: 5 questions). Answers to these questions were captured on an 11-point scale, with ratings from zero (none of the time [poor]) to 10 (all of the time [excellent]). Microsoft Excel was used to capture the data, which were then exported to SPSS, in which descriptive statistics were generated.

A 14-Day Discharge Survey (satisfaction survey) was also offered to patients 14 days after discharge from either GO or CR surgery to capture patients’ experiences with the ERAS app. The survey assessed satisfaction of the at-home postoperative program in the ERAS app. In the survey, 10 questions were asked to capture patient experience, usefulness of the app, the quality of app features, and suggestions for app improvement. Responses were exported into a Microsoft Excel spreadsheet and subsequently in SPSS, in which descriptive statistics were generated. Open-ended responses were themed in NVivo.

### Ethical Considerations

All activities were in accordance with the Guiding Principles for Evaluation [18]. Data were handled according to the guidelines outlined in the AHS document, Safeguarding Data for Evaluation and Quality Improvement [19], and by the rules set out in the Health Information Act [20].

In March 2018, the ERAS app for patients received ethics approval (REB17-1316) from the Conjoint Health Research Ethics Board from the University of Calgary. Ethics approval included the ability to recruit up to 125 participants in the study and targeted patients undergoing GO surgery and CR surgery. Activities included interviews, surveys, and accessing registries and databases. Informed consent was collected from all participants prior to data collection, and verbal consent to record interviews was also gathered. Data collection tools and their consent statements are included in the multimedia appendices.

Data were collected and analyzed by a team in AHS. Only this team had access to the raw data. Survey results, interview data, and data retrieved from the AHS Discharge Abstract Database were de-identified. Every effort was made to ensure participant anonymity. AHS retained all raw data in protected drives and password-protected files. Only aggregate level-results were shared. No compensation was provided to participants in this study.

## Results

### Patient Interviews and Satisfaction Survey

Of 102 users enrolled in the ERAS app, 47 patients completed the QoR-15 survey (40 GO and 7 CR surgery patients) [21] for a response rate of 42%. We interviewed 18 patients using the semistructured interview guide, and 46 patients completed the 14-Day Discharge Survey. Responses were analyzed into the following themes: (1) commendations, (2) frustrations, and (3) suggestions for improvement.

#### Commendations

Of the 18 interviewed patients, 10 patients (2 CR, 8 GO) reported the “What to Expect” feature was the most helpful feature in preparing them for surgery, and 6 patients (1 CR, 5 GO) used this feature for general “surgery information” and for “pre-surgery preparation.” In the 14-Day Discharge Survey, 13 patients (1 CR, 12 GO) also mentioned the “What to Expect” feature as the most helpful to them.

Patients felt the ERAS app helped to set expectations going into and out of surgery. One-half (9/18, 50%; 2 CR, 7 GO) of the interviewed patients used the app to self-monitor their recovery, and 6 patients (1 CR, 5 GO) used it to understand if their surgery experiences were normal. In the 14-Day Discharge Survey, 4 GO patients reported appreciating the video materials, which helped them prepare for surgery.

In addition, 5 interviewed patients (1 CR, 4 GO) found the ERAS app “reminders” helpful, 3 patients (1 CR, 2 GO) liked using the “Daily Health Checks,” and 2 GO patients found the “Checklists” useful. In the 14-Day Discharge Survey, 5 GO patients and 2 CR patients also liked the reminders. As reported on the 14-Day Discharge Survey, the “Daily Health Check” recommendations were helpful for 23 patients (1 CR, 22 GO), and 33 patients (5 CR, 28 GO) followed the app recommendations. Some patients (1 CR, 3 GO) who completed the 14-Day Discharge Survey found the “Checklist” helpful as well. Patients noted that the reminders, Daily Health Check, and checklist features covered questions and preparations they needed to know before surgery. All patients interviewed found the “initial sign-up” process of the ERAS app easy and straightforward. Although 2 GO patients reported that they were not verbally informed of the app, they were provided information about it with other handouts they received from the hospital.

Almost all (16/18, 89%) interviewed patients experienced no glitches when using the ERAS app. Only 2 patients (1 CR, 1 GO) experienced glitches such as the app crashing or the app not reappearing after crashing. No frustrations were experienced by 3 patients (2 CR, 1 GO) when using the app, and 3 GO patients were satisfied with the app and did not have any suggestions for improvement. Of the surveyed GO patients, 14 did not want to remove any questions from the Daily Health Check and found the questions sufficient.

Overall, most interviewed patients reported very positive experiences with the ERAS app. Patients thought the app was informative (1 CR, 3 GO), and 2 GO patients thought it was easy to use. One GO patient reported that the app reduced their anxiety and uncertainty around their surgery, and another said it benefitted their family by providing the necessary information for each stage of their surgery care. Some patients (2 CR, 12 GO) who completed the 14-Day Discharge Survey also had positive experiences with the app regarding its ease of use and engagement with their care.

The ERAS app had a positive impact on preparing patients and normalizing their recovery symptoms. All of the app features were appreciated and alleviated patients’ anxiety around their surgery procedure with reassurance they were on the correct path. The 14-Day Discharge Survey showed that the majority of both GO (28/40, 72%) and CR (6/7, 86%) surgery patients found the ERAS app helpful for their self-care at home. However, 11 of the 40 GO patients (28%) and 1 of the 7 CR patients (14%) did not find it helpful.

#### Frustrations

Frustrations with the app function refer to technological issues that could potentially be resolved by the ERAS app developer team. Of the interviewed GO patients, 3 felt that, without their medical background, the information in the ERAS app would be “too advanced for them.” Another 3 GO patients felt they received “too many app reminders” especially after their recovery improved. Additionally, 1 GO patient could not check items off their app to-do list, while another felt the list was too generic.

Textbox 1 lists the challenges with the ERAS app functionality expressed by 6 interviewed and 4 surveyed GO patients.

**Challenges with the Enhanced Recovery After Surgery (ERAS) app functionality expressed from January 2019 to October 2019 by gynecologic oncology patients from the Calgary’s Foothills Medical Centre (n=15), evaluated using a sequential mixed methods exploratory strategy.**

1. Too many repetitive questions and reminders
2. Unable to contact support from a phone number provided in the app
3. ERAS app timing out too quickly during short inactivity while doing the Health Check survey
4. Not knowing how long the ERAS app would be accessible to patients
5. ERAS app not feeling tailored to patient surgery type
6. Having to create too many accounts to get it working or issues with logging into the app
7. Inability to make changes or add free text responses (to say “I’m fine” in the Health Check survey if a question does not apply [ie, dehydration section]), report “no changes,” or change surgery dates and survey responses)
8. Inability to input responses on behalf of the patient

App design frustrations refer to ERAS app content issues that could potentially be resolved by the app information team. Of the interviewed GO patients, 2 reported that the ERAS app information provided was too generic. Furthermore, another interviewed GO patient and 1 GO patient who completed the 14-Day Discharge Survey found the information mostly applied to an average healthy patient and would be less helpful for patients who do not fit that profile. Another GO patient who was interviewed and 1 GO patient who completed the 14-Day Discharge Survey requested more age-appropriate wording (eg “bowel movement” instead of “poo” and “urination” instead of “pee”)*.*

Two interviewed GO patients and 1 surveyed GO patient wanted more specific information on bleeding, a GYN category, and other languages to accommodate non-English speakers. One interviewed CR patient requested more specific information on cramps. Another surveyed CR patient suggested specific pre-populated answer suggestions for questions about their bowel movements.

Four interviewed GO patients and 1 surveyed GO patient did not consistently use the ERAS app because they were in too much pain after going home from the hospital, were too sedated in the hospital, or did not think the app guidelines were applicable to them; they also did not use it until after their surgery because their surgery occurred soon after their referral.

Three interviewed GO patients and 1 surveyed CR patient expressed frustrations about health care staff being unaware of the ERAS app when they tried to talk to staff about or had questions regarding the app. Another GO patient preferred to have an introduction to the app from staff.

Overall, although most patients generally had positive experiences with the ERAS app, most reported feeling frustrated with the lack of app personalization. Patients preferred more options to reflect how they felt and the ability to remove items that were not applicable to them. When patients had questions about the ERAS app, they also noticed that staff were unaware of the app.

#### Suggestions for Improvement

Of the interviewed GO patients, 3 felt that the app was not personalized to them or relevant to their surgery type. One interviewed GO patient preferred more question variety because they felt the questions were repetitive. Textbox 2 lists the improvements for the ERAS app technological functionality suggested by 5 interviewed and 4 surveyed GO patients.

**Suggestions to improve the Enhanced Recovery After Surgery (ERAS) app technological functionality expressed from January 2019 to October 2019 by gynecologic oncology (GO) patients from the Calgary’s Foothills Medical Centre (n=15) and colorectal (CR) patients from Edmonton’s Grey Nuns General Hospital (n=3), evaluated using a sequential mixed methods exploratory strategy.**

GO patients:

1. Notifying patients how long they will have access to the ERAS app
2. Ability to change surgery dates
3. In-app browsing for links
4. Food plans for after surgery
5. The ability to create custom reminders about needed items for surgery
6. Ensure patients have someone to help them with mobility
7. An in-app notetaking feature (eg, for noting recommendations or surgery experiences)
8. Clarity on whether to follow tasks listed under “view future tasks” as they are not in the Daily Health Check List
9. The option to have a caregiver fill in surveys if the patient is unable to do so

CR patients:

1. An option to unsubscribe from notifications
2. A comparison or summary feature that displays a table or graphic of the patient’s progress before surgery, the day of surgery, and after surgery
3. A table to display where a patient’s progress is expected to be on different days
4. A fix for the app crashing issues

Of the surveyed GO patients, 13 suggested 12 different items to be tracked in the app. These are listed in Textboxes 3 and 4. Regarding the suggestions for the ERAS app design, 2 interviewed GO patients wanted phone numbers to contact support staff about technological issues or for other inquiries that require a doctor or nurse (Textbox 2)*.* Two patients (1 CR, 1 GO) suggested gathering feedback from patients about their experience sooner after their recovery because 1 patient was contacted 6 weeks to 8 weeks after surgery. Five interviewed and 5 surveyed GO patients wanted the app to have more specific information on the items listed in Textbox 2*.*

**Recommendations to improve the Enhanced Recovery After Surgery (ERAS) app health functionality expressed from January 2019 to October 2019 by gynecologic oncology (GO) patients from the Calgary’s Foothills Medical Centre (n=18) and colorectal (CR) patients from Edmonton’s Grey Nuns General Hospital (n=4), evaluated using a sequential mixed methods exploratory strategy.**

GO patients

1. Examples of appropriate or inappropriate activities at different stages of recovery (eg, how much walking or sitting one does)
2. How to cope with pain, headaches, and fatigue; understanding the different types of pain
3. Days bowel movements occurred and days of vaginal bleeding
4. Visual day-to-day tracking of progress
5. Creating and tracking of health goals
6. Progression of incision or staple recovery
7. Amount of sleep per day (eg, too much, adequate, too little)
8. Tracking of weight

CR patients

1. Days of bowel movements
2. Exercise plans
3. Diabetes diet plan
4. Healthy food intake

**Recommendations to improve general Enhanced Recovery After Surgery (ERAS) app functionality expressed from January 2019 to October 2019 by gynecologic oncology (GO) patients from the Calgary’s Foothills Medical Centre (n=18), evaluated using a sequential mixed methods exploratory strategy.**

1. A GYN category
2. Abdominal or knee surgery
3. “Other” complications
4. Real-world activities a patient should be able to perform at different stages
5. Movement limitations when patients return home
6. Common concerns from patients (eg, back aches, amount of pain experienced, heart burn, side effects like gas or chills, how and when to decrease pain medications, incisions, hematomas)
7. Breathing activities or instructions
8. Self-administered pain killers
9. Substitutes for people with diabetes
10. How patient app information is being used
11. The “What to expect” section
12. More detailed descriptions
13. Reminders not to chew gum or eat hard candy postsurgery
14. More examples of healthy food choices and education regarding healthy eating after surgery to support recovery

In summary, many patients suggested features that would provide them with more control and make the ERAS app more personalized to their experience. The ability to remove redundant information, reduce reminders, and track more health categories were important to patients.

All interviewed patients would recommend the ERAS app to a friend or family member because of the ease of access to surgery information and managing their surgery journey. One interviewed GO patient had already recommended it to a friend going for surgery, and another interviewed CR patient had mentioned it to a friend. One interviewed GO patient mentioned that they did not use the app much because their complications were beyond what the app could address. Another interviewed GO patient found the information in the app too generic. The 14-Day Discharge Survey results also demonstrated that patients from both the GO (36/40, 92%) and CR (7/7, 100%) groups would recommend the ERAS app to others. Only 1 (1/40, 8%) GO surgery patient would not.

### Quality of Recovery—Patient Outcomes

The QoR-15 questionnaire was administered to GO and CR patients 14 days after their surgeries to capture their general well-being for the past 24 hours (10 questions) and if they felt pain, nausea, anxiety, or depression in the last 24 hours (5 questions).

Overall, GO and CR surgery groups rated “communication with friends and family” (9.38/10), “personal toilet and hygiene unaided” (9.15/10), and “breathing easy” (8.94/10) in the last 24 hours (14 days postsurgery) the highest. The lowest scores were reported for having “good sleep” (7.00/10), receiving “support from health care team” (6.26/10), and “returning to work” or “usual home activities” (4.43/10) in the last 24 hours.

Overall, 14-day discharge data from ERAS app patients who completed the QoR-15 survey (Part A) showed the highest ratings for communication with friends and family (9.38/10), personal toilet and hygiene unaided (9.15/10), and breathing easy (9.94/10). The highest score from the QoR-15 survey (Part B) was for moderate pain (3.79/10); however, this is still a low score out of 10.

**Figure 2:**
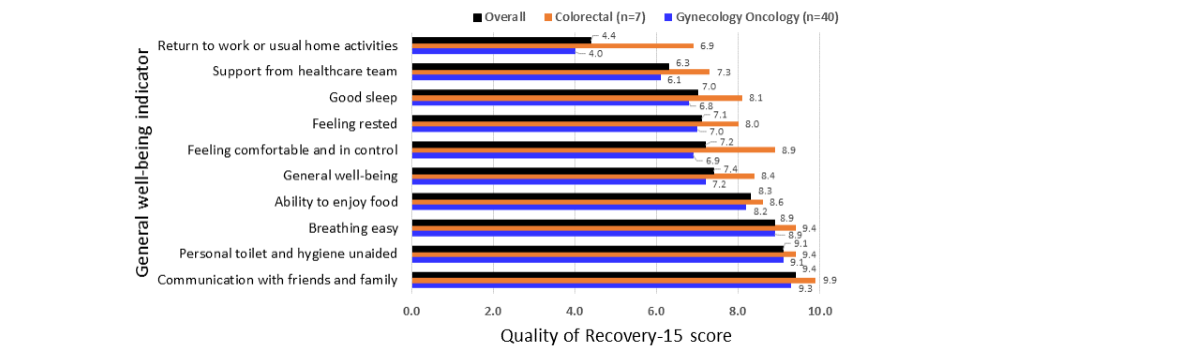
Quality of Recovery-15 scores for general well-being with the Enhanced Recovery After Surgery (ERAS) app.

The lowest QoR-15 (Part A) survey scores were shown for good sleep (7.00/10), support from health care team (6.26/10), and returning to work or usual home activities (4.43/10). Although sleep and support from the health care team were some of the lowest scores, overall, they were still high scores for patient health (Figure 2). The lowest score for returning to work or usual home activities could be due to patients naturally needing time off to recover from surgery. The lowest scores from the QoR-15 survey (Part B) were severe pain (0.70/10) and nausea or vomiting (0.60/10), meaning that they were experienced infrequently (with a top score being 10).

Patients were asked to answer 5 questions about what they experienced in the last 24 hours (14 days postsurgery). Each question provided an 11-point scale from zero (none of the time [poor]) to 10 (all the time [excellent]). Only data from patients discharged after 14 days were used.

**Figure 3:**
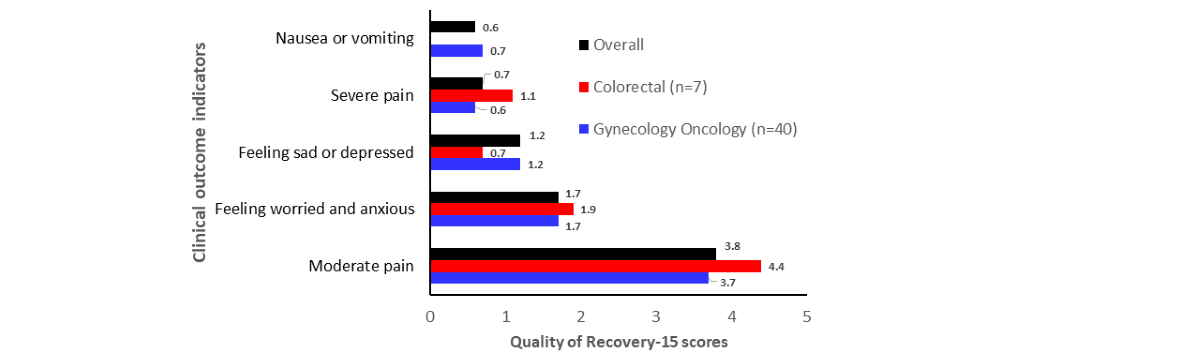
Quality of Recovery-15 scores for clinical outcomes with the Enhanced Recovery After Surgery (ERAS) app.

With regards to the 5 questions in the QoR-15 survey on pain, nausea, anxiety, or depression, GO and CR surgery groups showed low ratings overall, but the highest ratings were for moderate pain (3.79) in the last 24 hours (Figure 3)*.* The lowest scores were for having severe pain (0.70) and nausea or vomiting (0.60) in the last 24 hours. Ratings were consistently lower (out of 10) for ERAS app GO patients compared with CR patients (Figure 3)*.* The range of differences in the ratings across questions between the GO and CR surgery groups was 0.33 to 2.86 out of 10. GO and CR surgery groups were similar in their ratings for breathing easy, ability to enjoy food, personal toilet and hygiene, and communication with friends and family in the last 24 hours (14-days postsurgery; within 0.6 of 10).

Ratings varied across the 5 clinical questions between the ERAS app GO and CR patients (Figure 3)*.* The range of differences in the ratings across questions was 0.18 to 0.75 out of 5. GO and CR surgery groups were similar in their ratings for feeling worried or anxious in the last 24 hours (14 days postsurgery; within 0.18 of 10). The CR surgery group had higher ratings for moderate pain and severe pain, and the GO surgery group had higher ratings for nausea or vomiting and feeling sad or depressed in the last 24 hours (14 days postsurgery).

### Clinician Interviews

In total, 8 of the 22 clinicians involved in this study agreed to participate in an interview using a semistructured interview guide, with 2 clinicians who responded by email. One clinician emphasized the potential of the ERAS app to give providers insight into patient knowledge gaps and opportunities to improve on that knowledge. Although clinicians did not think it was a barrier overall, 1 clinician would have preferred more information on how best to recruit patients, and another clinician desired the ERAS app to reach its full operational capability sooner.

As part of the study, preoperative clinic staff were tasked with introducing patients to the ERAS app and informing them about online self-registration. One-half (4/8, 50%) of the clinicians had positive experiences recruiting staff and patients to engage with the app, indicating that flyers and handouts supported recruitment. Furthermore, clinicians reported that having a hands-on experience (eg, testing out a demo) with the app increased clinician buy-in and promotion of the app to patients. Clinicians had a positive experience recruiting staff and patients due to a more streamlined sign-up process and greater involvement with the ERAS app and its promotion.

Of the clinicians, 25% (2/8) reemphasized that patients would need hands-on familiarity with the ERAS app to increase buy-in. Another 2 (25%) clinicians found that the recruitment was slow because the app rollout was in a research phase and some staff were not promoting the app to patients but rather including the app information with other handouts to patients. Additionally, clinicians indicated it was easier to sign up for the app on a desktop computer rather than on a smartphone, which may have hindered enrollment. Another clinician thought that the app should be introduced to patients during surgical discussions and again before they discharge.

Clinicians reported easier implementation, as the ERAS app involved their input and provided more clarity to other staff members involved. Of the 8 clinicians interviewed, 3 discussed the ERAS app’s implementation. Generally, clinicians felt that the implementation was successful but suggested some areas for improvement. One clinician (1/8, 13%) reported that the app would not apply to every patient but will likely be applicable to most patients. One clinician stated that implementation was much “smoother” and provided greater clarity on medical information because it incorporated health care workers’ feedback. One clinician expressed concern that involving too many members of the leadership team in implementation might result in delays to the ERAS app’s release and would potentially exclude some patients due to a late implementation.

All clinicians had positive experiences with the ERAS app, as they experienced its potential to decrease unnecessary visits and phone calls to reassure patients. Clinicians were concerned that the app may not be beneficial to patients with specific needs and non-English speakers. All clinicians reported positive experiences with the ERAS app, and the vast majority (7/8, 88%) received positive feedback from patients about the app. Specifically, clinicians appreciated the way the app (1) redirected the patient to the nurses when it required their intervention, (2) was easy to navigate, (3) was simple for older adults to use, (4) emphasized accessibility and made pre and postoperation information available to the patient, and (5) had improved overall since its initial rollout.

Clinicians further elaborated on how the ERAS app benefited providers. Many clinicians (3/8, 38%) appreciated that the app provided medical information consistent with their recommendations. Other factors that clinicians identified as benefits were the following: (1) other staff members liking the ERAS app and (2) flyers and handouts that made introducing the ERAS app less intrusive to patients and saved provider time with explanations. Clinicians described the potential of the ERAS app to benefit the health care system. One-half of the clinicians (4/8, 50%) thought the app could help decrease unnecessary visits and calls from patients who need reassurance from a provider, as those concerns could be handled by the app instead. Some clinicians (3/8, 38%) also thought the app could make surgical information and directions easier for patients to follow and agreed that it could generally help the health care system by reducing that burden on specialists. Additionally, clinicians perceived multiple other potential health care system benefits could be attained by using the app. These are listed in Textbox 5*.*

**Clinician-identified potential health care system benefits with the Enhanced Recovery After Surgery (ERAS) app, as reported from January 2019 to October 2019 by 8 clinicians from Calgary’s Foothills Medical Centre and Edmonton’s Grey Nuns General Hospital and analyzed using a sequential mixed methods exploratory strategy.**

1. Expanding the ERAS app to other health complications
2. Accelerating patients’ discharge, since they can receive the same care advice via the ERAS app as they would get in the hospital
3. Increasing patients’ absorption of care information, as they receive it through the ERAS app when it is relevant
4. Helping cancer patients keep track of being on blood thinners
5. Normalizing patients’ experiences, so they understand that most of their symptoms are typical
6. Providing opportunities to compare operative reports for cancer surgery between Edmonton and Calgary

Although most clinicians (7/8, 88%) were satisfied with the ERAS app, some expressed challenges, particularly around responding to patients’ specific technological needs. Several clinicians (3/8, 38%) expressed concern that older patients and others who were not technologically savvy may have trouble using the app. Other clinicians (2/8, 25%) noted the app posed a language barrier for non-English speakers, and general app information for an average patient led to confusion for patients who had specific needs. Additional challenges, as well as technological challenges, noted by clinicians are listed in Textbox 6.

**Clinician-identified challenges, including technological challenges, with the Enhanced Recovery After Surgery (ERAS) app, as reported from January 2019 to October 2019 by 8 clinicians from Calgary’s Foothills Medical Centre and Edmonton’s Grey Nuns General Hospital and analyzed using a sequential mixed methods exploratory strategy.**

Challenges

1. Lack of clinical time to introduce the ERAS app
2. Frustration that an ERAS app is overdue, as other professions have similar technologies
3. Potential lack of complete applicability to patients with diabetes (eg, the effect of carbohydrate loading on diabetes) or other complex cases
4. The need to explore why some physicians are not using the ERAS app (lack of buy-in)
5. A potential overestimation of patients with wound infections (if they are redirected to their general practitioner and not a wound care clinic)

Technological challenges

1. No option for patients to upload and send a picture of their wound
2. Difficulties switching from email to phone notifications
3. One patient declining to participate because their phone was incompatible with the ERAS app
4. Many older patients not having an iCloud account set up on their iPhone, so they could not download the app

Most clinicians (7/8, 88%) believed that the ERAS app should be continued and expanded. A couple of clinicians (2/8, 25%) wanted the app to be expanded to other surgery types to help address gaps in patient education. Another clinician suggested creating a short video to demonstrate the app and its functions.

### Patient Acceptance

Patients who used the ERAS app and participated in the evaluation generally found the app to be helpful in their surgery preparation and recovery. The app improved patients’ access to reliable objective information regarding their surgery, provided them with clarity about all stages of their surgery experience, and supported self-management of their care. Surgery patients may contact their nurse or surgeon for clarification and reassurance in what they perceived as abnormal recovery symptoms from the surgery. Patients may need this reassurance and clarification from clinical staff because of trust in their expertise. The app appeared to reduce some of those calls and gave patients clinician-trusted support through the app while concurrently reducing the dependency on clinicians as their first point of contact for reassurance concerns.

The app is a dependable digital tool that empowered patients to keep track of their recovery by using the app’s Daily Health Checks, checklists, and reminders. These app features helped cover questions and preparation items that are important to the patient journey to full recovery and addressed patients’ knowledge concerns. Based on their positive and smooth experience, patients highly recommended the app, which not only made it easy for most patients to sign up but also functioned with minimally reported technological glitches and provided a seamless experience for those who were not technologically savvy. Thus, it is not surprising that all patients who participated in the evaluation would recommend the app to a friend of family member. Most positive patient feedback remained the same throughout implementation. An improved experience reported later in implementation involved the ERAS app sign-up procedure, which was confusing and unclear for both clinicians and patients at the beginning but became easy and straightforward for all patients later. This likely improved due to allowing patients to sign up themselves through a referral code and removing the overreliance on clinicians and other health care staff to gain access to the app.

This study design provided an excellent opportunity to gather rich feedback for improvement from patients. An example of valuable feedback from patients with a medical background is that some information is too technical and that there is a need for some patient input in refining the language to a grade level of comprehension that the average patient could understand, thus making the app more inclusive and accessible. This could avoid making patients feel overwhelmed, anxious, and confused. Another important lesson learned during this study is to be mindful of the fact that the immediate postsurgical recovery period is sometimes quite difficult for patients and that not all patients are receptive to the constant reminders and check-ins, thus having a quite opposite effect than intended. The study also revealed some significant communication issues within the system, highlighted by the fact that some health care staff were not aware of the implementation of the app, such that when some confused patients reached out to nurses for clarification, they could not get the help for which they were looking. Some patients were unable to consistently use the ERAS app due to recovery from their surgery. Others were in too much pain or too sedated after their surgery to use the app; however, most of these patients used it before their surgery or after they started to feel better. The patient-reported quality of recovery outcomes showed that the app helped with communication with friends and family, personal hygiene, and breathing easy.

Although it is clear that the concept of a digital app could help patients in their surgical journeys, it is important to strike a healthy balance between standardizing the pathways and customizing a few elements of the app for some patient populations.

### Clinician Acceptance

Clinicians who participated in the evaluation reported having a positive experience with the ERAS app and received positive feedback from patients. Clinicians appreciated the simplicity and consistency of the medical information, similar to what they would recommend to patients. This could help clinicians feel confident that patients are following medical advice consistent with best practices and recommendations from their team. Clinicians reported that the app was simple and intuitive enough so that older adults were comfortable using it. Including clinician input and involvement helped boost clinician confidence and ownership of the app. Doing so may have further supported clinician buy-in and greater promotion of the app among patients and colleagues. Most clinicians were busy, so clear communication and an unobtrusive merging of a new app into their practice was important to them. Clinicians recommended continuing with and expanding the app to other surgery types.

Clinicians also noted that the staff needed hands-on familiarity to increase buy-in and to help introduce the app to patients. Some staff members were unaware of the app, did not introduce it to patients, and potentially missed out on additional patient recruitment. It was suggested that, if staff had hands-on familiarity with the app, they would see the value in it and be able to inform patients about its benefits due to their firsthand experience.

Clinicians noticed that some app information may not be applicable to patients with complex health conditions or non-English speakers. Similarly, clinicians noticed that the information applies to the vast majority of, but not all, patients. The app could consider patient complexity and identify areas or patients for which it may not be completely beneficial. Adapting the app to other languages may expand the number of patients who could benefit from it.

Clinicians experienced more improvements as implementation progressed. Clinicians noted how streamlined the recruitment process became with the creation of handouts and flyers and allowing patients to self-sign up to the app. In the early implementation, clinicians also were concerned that patients who were older may have difficulty using the app, but as implementation progressed, it was observed that mostly older patients were using the app with few difficulties.

This evaluation provides valuable feedback from users on the acceptability of the ERAS app. It was a useful tool to support patients in their surgery preparation and recovery as well as for clinicians.

## Discussion

### Principal Findings

Compared with other medical interventions, surgical care can inherently carry significantly higher risks. This is stressful for patients (experience and clinical outcomes), providers, and the overall health care system. A strategy to alleviate these stressors is to put in place systems, protocols, pathways, guidelines, and mechanisms to assist patients throughout the entire surgical journey, starting with the decision to treat by surgery, followed by the time spent in hospital and the postoperative care and transition back into the community. The ERAS pathway in one such clear example that is in line with the Home to Hospital to Home concept [22]. A pathway is effective only if there is adherence to the prescribed recommendations by patients and compliance with the established checks and balances by health care providers. However, it is well documented in the literature that, in the busy health care environment and patients’ hectic lifestyles, it is not always possible to keep track of all preparations before surgery and the postoperative actions that will mitigate the risk of complications and enhance recovery [23].

In the current digital age, in which patients and providers in developed countries have near-24/7 access to the internet, it is worthwhile to leverage digital technologies to ensure that patients have access to high-quality, unbiased sources of information and effective means to keep patients on track on their surgical journey. This exploratory research on the use of the ERAS app by CR and GO patients indicates a high level of acceptance by both patients and providers. This finding is not different from other studies investigating the use of apps for cancer care [24], diabetes management [25], kidney disease [26], or chronic pain [27]. More importantly, another study by Schlund et al [28] looking at the impact of the ERAS app showed improved medication adherence, fewer surgical site infections, and decreased cost for the health care system. Compared with other studies, we went one step further and elicited input from both patients and providers on how to improve the interactivity and functionality of the ERAS app. The findings of this exploratory study are timely in the current post-COVID-19 period in which digital health is quickly becoming the norm and during these trying times of unacceptably long surgical wait times. Further studies with robust study designs are needed to confirm whether the use of the ERAS app can have potential downstream positive impacts on health system utilization.

### Limitations of the Study

Although this study is a reasonable attempt to test the implementation of an app to accompany patients in their surgical journey, it does have several limitations that have to be considered when attempting to scale and spread across the health care system. First, due to funding limitations, the study was limited to only 2 surgical specialties, namely CR and GO surgery. Second, the implementation of the ERAS app was limited to only 2 major urban hospitals; thus, the findings cannot be extrapolated to low-volume and rural hospitals. Additionally, there were fewer patients recruited overall, resulting in potential selection bias. The perspectives of patients who stopped using the app and did not complete the study were not investigated. Third, the small sample size of patients may not be representative of the larger population. Therefore, the results should be examined as informational about the experience with the app. Fourth, given their heavy workload, we were able to interview only 4 surgeons to get their in-depth feedback on the performance of the app. Finally, interview questions were subject to participant interpretation. Questions were amended, and interviewers probed when necessary to ensure clarity and understanding of clinicians and patients. The small sample size also restricted the ability to stratify patient interview results by surgery type or clinician interview by role; therefore, results were aggregated for reporting. Finally, in general, CR surgery patients at GNCH had lower enrollment than GO patients at FMC. This discrepancy is likely due to a lower number of CR surgeries performed and a lower volume of patients at GNCH. Enrollment activities were the same at both sites.

### Conclusion

Our study, which pre-dates the COVID-19 pandemic, is an important proof-of-concept study that highlights the importance of using digital technology to improve patient and provider experiences. With some customization and refinements, this app has the potential to improve patient satisfaction and provider experience. Patient experience ratings, including support, were high, which could lead to better recovery and fewer primary care follow-up visits (an outcome not explored in this study). Further studies, with robust study designs using concepts and models of implementation science, are warranted to confirm our findings before wider adoption of the app in surgical pathways. Patient and provider experiences were a valuable source of feedback for the evaluation of this type of app. Widely adopted during the pandemic, digital health technologies are here to stay for the long term. This is a unique opportunity for policymakers to rethink their strategies to adopt digital technologies to improve the performance of their health systems and make them more resilient.

## Appendix

Multimedia Appendix 1 Clinician consent form.

Multimedia Appendix 2 Interview guides.

Multimedia Appendix 3 Surveys.

## Abbreviations

AHS: Alberta Health Services
CR: colorectal
ERAS: Enhanced Recovery After Surgery
FMC: Foothills Medical Centre
GNCH: Grey Nuns Community Hospital
GO: gynecologic oncology
QoR-15: Quality of Recovery-15

## Data Availability

Data collected were safeguarded and protected in secure Alberta Health Services data drives and are not publicly available. Correspondence with the primary author (SB) is recommended for any data-related inquiries: Sanjay.Beesoon@albertahealthservices.ca.
