# Supplementary material for: Patient and Provider Experiences With a Digital App to Improve Compliance With Enhanced Recovery After Surgery (ERAS) Protocols: Mixed Methods Evaluation of a Canadian Experience
Source: JMIR Form Res. 2023 Dec 15;7:e49277. doi: 10.2196/49277 (PMC10757223; doi:10.2196/49277)
Supplement: Multimedia Appendix 1 [file formative_v7i1e49277_app1.docx]

### Clinician Consent Form

**Introduction**

Thank you for your interest in participating in this interview. We sincerely appreciate your time.

**Purpose**

The overall goal is to hear about your experience with the **ERAS App**. In particular, we are interested in your views about what has been working well, what are some challenges experienced, and what are some improvements that you think may help service delivery. Your feedback will be used to inform App improvement for Phase II implementation. The interview will be about 15-30 minutes in length. Interview results will be compiled into a report and delivered to the ERAS Team.

**Consent**

Before agreeing to participate in the interview, you should know that:

- Participation is voluntary. You have the right to withdraw from this interview at any time without an explanation. If you decline to participate or withdraw, your current and future employment status will not be affected.
- Access to the notes taken and transcription of this interview will be limited to the staff at **Health Systems Evaluation and Evidence** tasked with capturing and analyzing the information.
- We will use direct quotes in the evaluation report; however, you will not be personally identified in any report. We will only use quotes that ensure your anonymity as a respondent.
- I would like to audio-record the interview to ensure accuracy of my notes. A copy of the interview notes and transcription can be shared with you upon request.
- All information used to inform this evaluation will be stored in a secure location and archived for five years. Only the evaluation team will have access to the original data.
- Do you have any questions?
  - If participant has questions about the evaluation that the interviewer is not able to address. Inform them to contact the Senior Consultant leading the evaluation [Ashley.Drobot@albertahealthservices.ca](mailto:Ashley.Drobot@albertahealthservices.ca) or (780) 407-4880

Are you willing to participate in this interview and have your answers included in the evaluation reporting?

___Yes – proceed.

___No – explore what would be required in order to proceed. Do not interview without explicit consent.

May I record our conversation?

___Yes – proceed with interview.

___No – do not record interview. Take as many verbatim notes as possible.
