# Supplementary material for: Patient and Provider Experiences With a Digital App to Improve Compliance With Enhanced Recovery After Surgery (ERAS) Protocols: Mixed Methods Evaluation of a Canadian Experience
Source: JMIR Form Res. 2023 Dec 15;7:e49277. doi: 10.2196/49277 (PMC10757223; doi:10.2196/49277)
Supplement: Multimedia Appendix 2 [file formative_v7i1e49277_app2.docx]

### Patient Interview Guide

Time duration: estimate 15 minutes.

Thank you for agreeing to this short interview and for using the ERAS App. Your input is very important as you were one of the first to use this App and we very much want to learn about your experiences, how it may or may not have helped you and what if any changes you would suggest. Please know that your responses will be grouped with those of other users and that your individual comments will not be identified with your name. Also, you may end this interview at any time and this not impact your care.

In order for me to ensure that I accurately collect your input, I would like to record this interview and then type out your responses. The audio tape will then be destroyed.

May I record our conversation? _ Thank-you.

1. What would you say was your overall experience like when using this App?

2. How did the recruitment and sign on process go? Did you experience any glitches or frustrations?

3. Can you share with me how you used the App?

4. Were there any times when it was particularly useful? Frustrating?

5. Would you recommend it to a friend or family member?

6. Do you have any suggestions?

Thank you so much for your time and for sharing your experiences. Your responses will be typed out and then this tape destroyed. Your responses will be grouped with those of other users and we'll share the findings with the App developers and the medical teams so that we can make this an even better experience.

Thank you again.

### Clinician Interview Guide

*I’d like to start by talking generally about the ERAs App.*

1. Overall how was your experience with the ERAS App pilot?
2. Were there any particular strengths or weakness from your perspective? From the patients’ perspective?
3. Do you have any comments or recommendations on how future Apps should be introduced so that both patients and clinicians feel supported in their adoption? Any implementation concerns?
4. Would you recommend that this App be continued? Expanded? And why or why not?
5. Any other comments which you would like to share with the App software developer or with the people who plan the implementation of any future roll outs?
