# Supplementary material for: Patient and Provider Experiences With a Digital App to Improve Compliance With Enhanced Recovery After Surgery (ERAS) Protocols: Mixed Methods Evaluation of a Canadian Experience
Source: JMIR Form Res. 2023 Dec 15;7:e49277. doi: 10.2196/49277 (PMC10757223; doi:10.2196/49277)
Supplement: Multimedia Appendix 3 [file formative_v7i1e49277_app3.docx]

### QoR-15 Survey

**QoR-15 Patient Survey**

*PART A*

***How have you been feeling in the last 24 hours?***

(0 to 10, where: 0 = none of the time [poor] and 10 = all of the time [excellent])

1. Able to breathe easily None of All of

the time 0 1 2 3 4 5 6 7 8 9 10 the time

1. Been able to enjoy food None of All of

the time 0 1 2 3 4 5 6 7 8 9 10 the time

1. Feeling rested None of All of

the time 0 1 2 3 4 5 6 7 8 9 10 the time

1. Have had a good sleep None of All of

the time 0 1 2 3 4 5 6 7 8 9 10 the time

1. Able to look after personal None of All of

toilet and hygiene unaided the time 0 1 2 3 4 5 6 7 8 9 10 the time

1. Able to communicate with None of All of

family or friends the time 0 1 2 3 4 5 6 7 8 9 10 the time

1. Getting support from hospital None of All of

doctors and nurses the time 0 1 2 3 4 5 6 7 8 9 10 the time

1. Able to return to work or None of All of

usual home activities the time 0 1 2 3 4 5 6 7 8 9 10 the time

1. Feeling comfortable and in None of All of

control the time 0 1 2 3 4 5 6 7 8 9 10 the time

1. Having a feeling of general None of A ll of

Well-being the time 0 1 2 3 4 5 6 7 8 9 10 the time

*PART B*

***Have you had any of the following in the last 24 hours?***

(10 to 0, where: 10 = none of the time [excellent] and 0 = all of the time [poor])

1. Moderate pain None of All of

the time 10 9 8 7 6 5 4 3 2 1 0 the time

1. Severe pain None of All of

the time 10 9 8 7 6 5 4 3 2 1 0 the time

1. Nausea or vomiting None of All of

the time 10 9 8 7 6 5 4 3 2 1 0 the time

1. Feeling worried or anxious None of All of

the time 10 9 8 7 6 5 4 3 2 1 0 the time

1. Feeling sad or depressed None of All of

the time 10 9 8 7 6 5 4 3 2 1 0 the time

### SeamlessMD Satisfaction Survey

SeamlessMD_AHS – 7 Days After Discharge Sat. Survey **2018**

**Alberta Health Services**

Procedure - Published - v.1 - 7 DAYS AFTER DISCHARGE - Satisfaction of the at-home after surgery program.

**Question 1:** Thank you for using SeamlessMD! Please take a few minutes to tell us about your experience. Your feedback will help us provide a better experience to future patients!

**Question 2:** How useful is SeamlessMD in helping you manage your self-care at home?

**Question 3:** How can we make SeamlessMD more helpful for patients at home?

**Question 4:** Would you recommend SeamlessMD to other patients having this surgery?

**Question 5:** What did you like best about SeamlessMD?

**Question 6:** What questions should we change or take out of the Health Check?

**Question 7:** What would you be interested in tracking every day on the Health Check?

**Question 8:** Did you find the recommendations in the Health Check helpful? Why or why not?

**Question 9:** Did you follow the recommendations in the Health Check? Why or why not?

**Question 10:** Is there anything we should add to the patient education library?

**Question 11:** Do you have any other comments to make SeamlessMD better for future patients?
